# Vasculoprotective effects of rosiglitazone through modulating renin-angiotensin system in vivo and vitro

**DOI:** 10.1186/1475-2840-10-10

**Published:** 2011-01-26

**Authors:** Liqun Ren, Naifeng Liu, Hong Zhi, Yingjuan Li, Yanzhi Li, Rining Tang, Zulong Sheng

**Affiliations:** 1 Department of Cardiology, Zhongda Hospital of Southeast University, Nanjing, China

## Abstract

**Background:**

The peroxisome proliferator-activated receptor-γ (PPARγ) agonist rosiglitazone has been suggested to exert cardiovascular protection through the improvement of lipid metabolism, anti-inflammation, anti-proliferation etc. However, whether renin-angiotensin system (RAS) is involved in the vascular protective effects of PPARγ agonists is not fully understood. The present study aimed to investigate the effects of the renin-angiotensin system in vascular protection mediated by PPARγ agonists.

**Objective:**

To investigate the actions of the renin-angiotensin system in vascular protection mediated by activation of PPARγ in vivo and in vitro.

**Methods:**

Rats were fed a regular diet (n = 8), a cholesterol-rich diet plus methylthiouracil (80 mg/Kg/day, n = 10), a cholesterol-rich diet plus methylthiouracil and rosiglitazone (4 mg/kg/day, n = 10). The rosiglitazone treatment was started from one month after the start of cholesterol-rich diet plus methylthiouracil, and lasted five months. Cultured vascular smooth muscle cells (VSMCs) were pretreated with 1 μmol/L angiotensin II (ANG II) for 6 h and randomly divided into the control group; the ANG II group (1 μmol/L ANG II); the groups respectively treated with different concentration rosiglitazone (20, 30, 50) μmol/L for 12 h; the groups treated with 30 μmol/L rosiglitazone for (6, 12, 24) h. Morphology changes of the aortic tissues were observed by hematoxylin and eosin stain. The VSMC growth was detected by 3-(4,5-dimethylthiazol-2-yl)-2,5-diphenyltetrazolium bromide (MTT) colorimetric assay. Angiotensin II and expression of angiotensin receptors were determined by radioimmunoassay, reverse transcription polymerase chain reaction (RT-PCR), western blot, and immunohistochemistry.

**Results:**

After 6 months, lipid deposition, VSMC proliferation and migration toward intima were observed in aortic tissues in the rats on a cholesterol-rich diet plus methylthiouracil, while these pathological changes induced by the cholesterol-rich diet were significantly suppressed by rosiglitazone. In addition, VSMC proliferation induced by ANG II was markedly inhibited by rosiglitazone. Rosiglitazone markedly down-regulated expression of angiotensin type 1 receptor (AT_1_R) and up-regulated expression of angiotensin type 2 receptor (AT_2_R) in the aortic tissues and ANG II-treated VSMCs.

**Conclusions:**

The present study demonstrated that PPARγ agonist rosiglitazone suppressed ANG II-induced VSMC proliferation in vitro and early atherosclerotic formation evoked by cholesterol-rich diet in vivo. These vasculoprotective effects of rosiglitazone were mediated at least partially by reduction in local tissue ANG II concentration, down-regulation of AT_1_R expression and up-regulation of AT_2_R expression both at the mRNA and protein levels.

## Background

Although meta-analyses on rosiglitazone have raised some concerns in its clinical use, there are no outcomes data to support these concerns. Peroxisome proliferator-activated receptor-γ (PPARγ), a member of the nuclear receptor superfamily of ligand-activated transcription factors, is a key regulator of adipogenesis and lipid metabolism [1]. Besides their well-recognized insulin-sensitizing property, synthetic PPAR-γ agonists, such as rosiglitazone, have been shown to possess strong anti-inflammatory properties [2]. In vitro, PPARγ agonist reduces intercellular adhesion molecule-1 (ICAM-1) expression in activated endothelial cells, inhibits production of proinflammatory cytokines (TNF-a, IL-6, and IL-1β) by activated monocytes, decreases transcription of monocyte chemoattractant protein, and significantly reduces monocyte/macrophage homing to atherosclerotic plaques [3-5]. In several studies, it has been demonstrated that treatment with PPARγ agonists markedly reduces MI/reperfusion injury in vivo, cardiac hypertrophy, and atherosclerotic lesion formation through anti- inflammatory effects [6-10].

Considerable evidence obtained from animal studies as well as clinical observations has demonstrated that hypercholesterolemia is an independent risk factor for coronary artery disease. Previous studies have also demonstrated that hypercholesterolemia is associated with an increased inflammatory response [11]. Moreover, hypercholesterolemia activates renin-angiotensin system (RAS) and accelerates atherosclerotic lesion formation [12]. Angiotensin II receptors have two main types that are called angiotensin II type 1 receptor (AT_1_R) and angiotensin II type 2 receptor (AT_2_R). Hypercholesterolemia increases AT_1 _receptor density and functional responsiveness [13,14]. More convincingly, atherosclerosis prone male apolipoprotein E (apoE) deficient mice that also lack the AT_1A _receptor (double knock out) exhibit reduced atherosclerosis compared with wild type apoE KO mice [15]. However, the interactions between the PPARγ activation and the RAS, which may have contributed to vascular protection against hypercholesterolemia, have not been previously defined. In addition, the role of the AT_2 _receptor in diet-induced hypercholesterolemia has been unknown.

Therefore, the aims of the present study were to investigate whether PPARγ ligand agonists exert vascular protective effects through the modulation of the RAS components, and if so, how--with particular regard to changes in angiotensin II receptor expression in the hypercholesterolemic rat model.

## Materials and methods

### In vivo studies

All procedures involving animals complied with national guidelines and were approved by the regional ethical committee. Male Wistar-Kyoto rats weighing 150-190 g and obtained from a professional provider (Si Lai Ke Experimental Animal Co. Ltd., Shanghai) were housed in a room with temperature maintained at 22°C and a light-controlled 12 h light/dark cycle. Water and food were freely available throughout the experiment. Twenty-eight rats were randomly allocated into one of the following groups: (1) the control group (Con group, n = 8), which was fed a regular diet; (2) the cholesterol-rich diet group (Cho group, n = 10), which was fed a cholesterol-rich diet plus methylthiouracil (80 mg/Kg/day); (3) the rosiglitazone group (Ros group, n = 10), which was fed a cholesterol-rich diet plus methylthiouracil and rosiglitazone (4 mg/kg/day; Shanghai Sunve Pharmaceutical Co. Ltd., China). The cholesterol-rich diet contained 10% lard, 4% cholesterol, 0.5% taurocholic acid, and 85.5% regular chow. Rosiglitazone or methylthiouracil was dissolved in 1 mL physiological saline and administrated once a day by gavages. The rosiglitazone treatment was started from one month after the start of cholesterol-rich diet plus methylthiouracil, and lasted five months.

### Morphology and immunohistochemistry

For each rat on three sections chosen from the aortic sample we performed hematoxylin and eosin (HE) staining and assessed aortic morphology changes. The vascular trees were isolated and perfused with phosphate-buffered solution (PBS) to clear the lumen of blood, followed by fixation with 4% paraformaldehyde for 5-10 min. The thoracic aorta was cleared from surrounding fat and tissue, and dissected along the long axis. Evaluation of atherosclerotic lesions was performed from longitudinal sections of aortic arches. A 3 mm long segment of the proximal aorta was fixed with 4% paraformaldehyde for 5 - 10 min and embedded in paraffin for later evaluation of lesion size (intima and media) and immunohistochemistry assays. The 4 μm thick serial sections were prepared and stained with HE for light microscopic evaluation. The lipid deposition in atherosclerotic lesions was visualized by oil red O staining as described previously [16]. The rest of the aorta was stored at -80°C for RT-PCR and western blot analysis of angiotensin II receptors, and for measurement of angiotensin II (ANG II).

Three sections chosen from aortic sample of per rat were performed immunohistochemical analysis for the expression of AT_1_R and AT_2_R. Immunostaining was performed using the avidin-biotin complex (ABC) method with horserasish peroxidase (HRP)-conjugated secondary antibodies and diaminobenzidine (DAB) as substrate. Briefly, 4 μm thick slides were dewaxed, rehydrated, air-dried, and blocked with blocking solution (H -1009, Sigma, USA). The sections were incubated at 4°C overnight with goat anti-rat AT_1_R or AT_2_R polyclonal antibody (cat no. sc-31181 and sc-48451, respectively; Santa Cruz Biotechnology, inc., USA) diluted 1:100 in 5% serum in PBS. Thereafter, the slides were incubated at room temperature for 90 min with an HRP-conjugated donkey anti-goat secondary antibody (AP180P; Pierce Biotechnology, USA) diluted 1:100 in 5% serum in PBS. Secondary antibody was detected with the ABC kit vectastain and DAB reagent (Vector Laboratories, inc.). Negative controls were run using an identical protocol but excluding the primary antibody.

Morphological differences were independently assessed in a blind fashion by two individuals who examined the same slides. Immunohistochemical analysis was performed using Olympus Micro Image analysis software (version 4.0; Olympus Optical, Japan). The positive staining areas were automatically traced. The total optical density (OD) was calculated using the following formula: OD = (1/red intensity + 1/blue intensity + 1/green intensity) × area of positive staining.

### In vitro studies

Vascular smooth muscle cells (VSMCs) were isolated from aortic media of four-week-old male Sprague-Dawley rats by enzymatic digestion and cultured in monolayer. Cultured VSMCs were confirmed by electron microscopy and immunocytochemical staining. VSMCs in passage 4 ~ 8 in log phase were used in following experiments. VSMCs were pretreated with 1 μmol/L ANG II for 6 h and randomly divided into the following groups: the control group (10% fetal bovine serum in Dulbecco's Modified Eagle Medium); the ANG II group (1 μmol/L ANG II); the groups respectively treated with different concentration rosiglitazone (20, 30, 50) μmol/L for 12 h; the groups respectively treated with 30 μmol/L rosiglitazone for (6, 12, 24) h. The VSMC growth was assessed by 3-(4,5-dimethylthiazol-2-yl)-2,5-diphenyltetrazolium bromide (MTT) colorimetric assay. mRNA and protein expression of angiotensin II receptors in all groups were detected by reverse transcription polymerase chain reaction (RT-PCR) and western blot, respectively.

### Biochemical analysis

Fasting serum lipid concentrations were determined using the cholesterol esterase/peroxidase enzymatic method for total cholesterol (TC), the lipase glycerol kinase enzymatic method for total triglycerides (TG), and the homogeneous assay for low-density lipoprotein cholesterol (LDL-C), as described in detail elsewhere and recommended by the National Cholesterol Education Program (NCEP), USA [17,18].

### Measurements of angiotensin II in plasma and aortic tissues

Prior to sacrifice of the Wistar-Kyoto rats at the end of 6 months of diet blood samples were taken by puncture of the left ventricular cavity. Aortic tissue was cut into tiny pieces and boiled for 15 min in 0.2 mol/L glacial acetic acid at the following ratio of tissue weight (g) to volume of glacial acetic acid (mL): 15:1. The homogenates of aortic tissue were centrifuged at 10,000 rpm for 15 min, at 4°C. Segregated supernatant fluid and plasma were frozen at -80°C for later measurement of ANG II concentration using radioimmunoassay (Beijing Atomic Hi-Tech Co., Ltd, China). Total protein in supernatant fluid was measured using the Coomassie Brilliant Blue method as described previously [19]. ANG II concentration in the tissues (ng per mg tissue) was calculated according to the formula: ANG II concentration (ng/mg) = ANG II in the supernatant fluid (ng/mL)/total protein in the supernatant fluid (mg/mL).

### Quantification of gene expression

Total RNA from the thoracic aorta or treated VSMCs was extracted using Trizol Reagent (Roche; Nanjing Bofei Biotechnology Co. Ltd, China), followed by reverse transcription according to the manufacturer's protocol. RT-PCR was performed according to standard procedures, with 35 cycles of amplification using primer sequences as follows: AT_1_R sense 5'- CTA CCG CCC TTC AGA TAA CA - 3' and antisense 5'- CCA AAT CCA TAC AGC CAC TC-3' (a 352-bp fragment); AT_2_R sense 5'- GGA CCT GTG ATG TGC AAA GT - 3' and antisense 5'- CAC GGG TAA TTC TGT TCT TC-3' (a 418-bp fragment). ß-actin (232-bp fragment) was selected as reference gene. Each run contained an internal control. The PCR reaction was carried out in standard buffer (TaKaRa Biotechnology Co. Ltd, Dalian, China). One-step RT-PCR conditions were as follows: AT_1_R: 1 min at 94°C, 45 seconds at 58°C, 1 min at 72°C; AT_2_R: 3 min at 94°C, 45 seconds at 56°C, 1 min at 72°C. The amplified products were separated on 2% agarose gels and analyzed using Gel-Pro analyzer software (version 3.0; Media Cybernetics, inc.). The density of each band was measured by densitometry. The values AT_1_R/β-actin and AT_2_R/β-actin were used to express the levels of AT_1_R and AT_2_R mRNA, respectively.

### Western blot analysis

The aortic tissues from four or five rats and treated VSMCs in each experimental group were analyzed in the western blots. Isolation of total protein, electrophoresis, and blotting were performed as previously described [20]. Briefly, equal amounts of membrane proteins (50 μg) from various groups were separated by 10% SDS-PAGE and transferred to polyvinylidene fluoride membrane (Pall Life Science, USA). After incubation in blocking solution (10% Bovine Serum Albumin) for 60 min, the membranes were incubated in a buffer containing 2.0 g/mL specific goat anti-rat AT_1_R or AT_2_R polyclonal antibody (1:1,000 dilution; Santa Cruz Biotechnology, USA), or β-actin antibody (1:8,000 dilution; Sigma). HRP-conjugated donkey anti-goat polyclonal antibody (Pierce Biotechnology, USA) was used as secondary antibody at 1:10,000 dilution. Chemiluminescent luminol reagent (sc - 2048, Santa Cruz Biotechnology, inc.) was used to detect the signal that was recorded on X-ray film. All western blot experiments were repeated at least three times with different aortic preparations. Band intensity was measured using Gel-Pro analyzer software.

### Statistical analysis

Statistical analysis was performed by using SPSS 12.0.1 software (SPSS, Inc., Chicago, IL). All data are expressed as mean ± SD. The differences between all measured values were assessed by one-way ANOVA followed by post-hoc analysis with Tukey multiple comparison test. A value of *p *< 0.05 was considered statistically significant.

## Results

### Angiotensin II in plasma and in aortic tissues

Plasma angiotensin II concentrations showed no significant differences between the groups. Angiotensin II in aortic tissues was substantially increased in the rats on a cholesterol-rich diet plus methylthiouracil (*P *< 0.01). Rosiglitazone markedly attenuated this increase in angiotensin II in aortic tissues (*P *< 0.01) (Table1).

**Table 1:** Comparisons of serum lipids, ANG II level in plasma and aortic tissues (mean ± SD)

groups	n	ANG II in plasma (pg/ml)	ANG II in aortic tissue (ng/mg)	TG mmol/L	TC mmol/L	LDL-C mmol/L
Con	8	142.61 ± 42.78	2.45 ± 0.10	0.79 ± 0.17	1.53 ± 0.23	0.31 ± 0.22
Cho	10	139.16 ± 64.46	4.72 ± 0.91 *	2.15 ± 0.30 *	4.66 ± 0.56 *	1.93 ± 0.21 *
Ros	10	173.97 ± 97.52	3.05 ± 0.39 ^‡^	0.58 ± 0.13 ^‡^	2.57 ± 0.27^‡^	1.20 ± 0.12 ^‡^

Con: regular diet group; Cho: cholesterol-rich diet plus methylthiouracil group; Ros: the group with cholesterol-rich diet plus methylthiouracil and rosiglitazone. *P < 0.01 versus Con group; ^‡^*P *< 0.01 versus Cho group.

### Lipid profiles

Taking the cholesterol-rich diet plus methylthiouracil for 6 months caused hypercholesterolemia, including increase in serum TC, TG, and LDL-C levels in 2 subgroups. However, the increases in TC, LDL-C, and TG levels were significantly suppressed in the rats on rosiglitazone treatment, as compared to those in the Cho group (*P *< 0.01) (Table 1).

### Evaluation of atherosclerotic lesions

**Figure 1:**
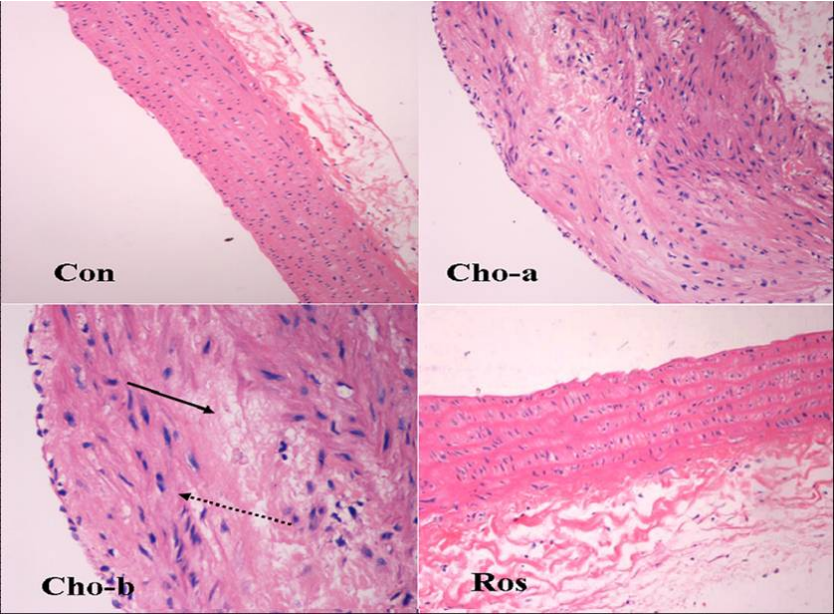
**Representative slides of hematoxylin-eosin staining of proximal aortic tissues**. Con: regular diet group (200 ×), showing integrated endothelial cell layer and regular arrangement of smooth muscle cells; Cho-a: cholesterol-rich diet plus methylthiouracil (80 mg/Kg/day) group (200 ×); Cho-b: cholesterol-rich diet plus methylthiouracil group (400 ×), showing smooth muscle cell proliferation, migration toward intima (broken arrow), and lipid deposition (solid arrow); Ros: cholesterol-rich diet plus methylthiouracil and rosiglitazone treatment (200 ×). Smooth muscle cell proliferation and lipid deposition were barely observed in the Ros group.

After 6 months in the Cho group, proximal aortas showed early arterial changes characterized by an accumulation of lipids in intima, VSMC proliferation, and migration toward intima, but no typical atherosclerotic plaques were found in any section of the proximal aorta. These pathological changes were barely visible in the rosiglitazone-treated rats (Figure 1).

### Evaluation of VSMC proliferation

**Figure 2:**
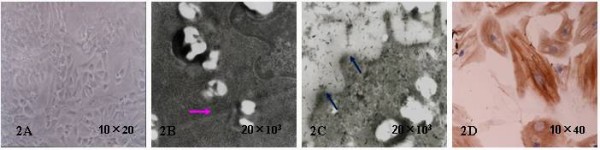
**In vitro cultured VSMC features**. Cultured VSMCs seen under in phase contrast microscope, were spindle or fusiform shape, showing valley-like feature (2A, 10×20); Unique cordlike filaments and macula densa in cytoplasm were observed under transmission electron microscope (2B and 2C, 20×10^3^); Immunocytochemical staining of VSMC α-actin clearly showed brown myofilaments of distribution along cell longitudinal axis. Cell nucleus were not stained (2D, 10×40).

Cultured VSMCs showed fusiform shape, valley-like feature under phase contrast microscope (Figure 2A). Unique cordlike myofilaments and macula densa were observed in cytoplasm under transmission electron microscope (Figures 2B and 2C). Myofilament structure of distribution along longitudinal axis were clearly visible, while cell nucleus were not stained (Figure 2D).

The mean absorbance in the VSMCs treated with 1 μmol/L ANG II for 6 h was significantly high as compared with that of the Con group (*P *< 0.01). The absorbance were markedly reduced in the VSMCs treated with different concentration rosiglitazones (20, 30, 50) μmol/L for 12 h or with 30 μmol/L rosiglitazone for (6, 12, 24) h, reaching a minimum in 50 μmol/L rosiglitazone for 12 h or 30 μmol/L rosiglitazone for 24 h respectively (*P *< 0.05, 0.01) (Tables 2 and 3).

**Table 2:** Comparisons of absorbance among groups treated with different concentration rosiglitazones for 12 h (mean ± SD)

groups	wells	Absorbance
control	3	0.25 ± 0.01
ANG II	3	0.51 ± 0.02 *
ANG II + 20 μmol/L Ros	3	0.48 ± 0.02 ^†^
ANG II + 30 μmol/L Ros	3	0.43 ± 0.02 ^‡^
ANG II + 50 μmol/L Ros	3	0.32 ± 0.01 ^‡^

*P < 0.01 versus control group; ^†^*P *< 0.05 versus ANG II group, ^‡^*P *< 0.01 versus ANG II group. Comparisons of among different concentration groups *P *< 0.05 or *P *< 0.01

**Table 3:** Comparisons of absorbance among groups treated with 30 μmol/L rosiglitazone for (6, 12, 24) h (mean ± SD)

groups		wells	Absorbance
control		3	0.23 ± 0.01
ANG II	6 h	3	0.52 ± 0.02*
ANG II + 30 μmol/L Ros	6 h	3	0.47 ± 0.02 ^‡^
	12 h	3	0.44 ± 0.02 ^‡ #^
	24 h	3	0.36 ± 0.03 ^‡ ##^

*P < 0.01 versus control group; ^†^*P *< 0.01 versus ANG II group; In rosiglitazone treatment groups, ^#^*P *< 0.05 versus previous point in time, ^##^*P *< 0.01 versus previous point in time.

### Protein and mRNA expression of angiotensin II receptors in aortic tissues

**Figure 3:**
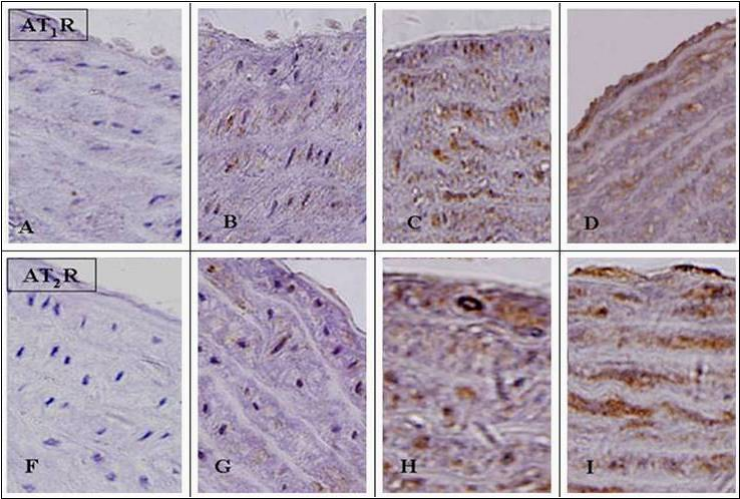
**Immunohistochemical staining of protein expression of angiotensin II type 1 receptor (AT**_**1**_**R) and angiotensin II type 2 receptor (AT**_**2**_**R) in aortic tissues**. A and F: the negative controls of AT_1_R and AT_2_R for the immunohistochemistry, respectively, showing no positive staining. B and G: the regular diet group; C and H: the cholesterol-rich diet plus methylthiouracil (80 mg/Kg/day) group; D and I: group with cholesterol-rich diet plus methylthiouracil and rosiglitazone treatment. Rosiglitazone treatment markedly attenuated AT_1_R immunoreactivity induced by the cholesterol-rich diet plus methylthiouracil (D), while increased AT_2_R immunoreactivity (I). (Magnification: 400×).

**Figure 4:**
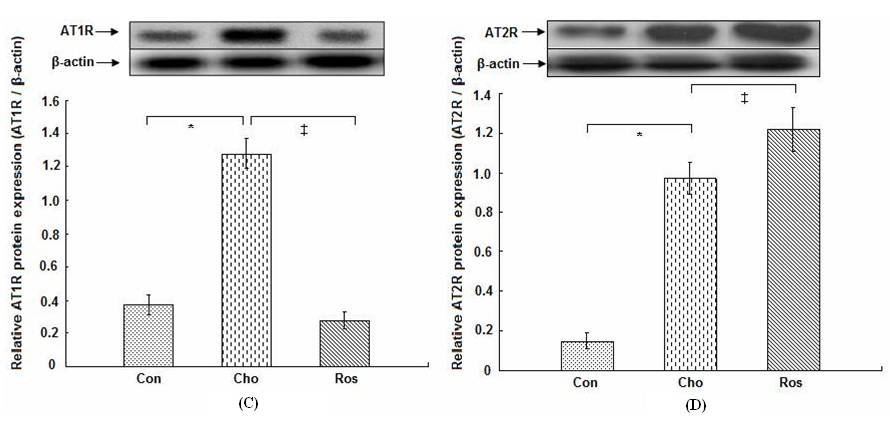
**Protein expression of angiotensin II type 1 receptor (C: AT**_**1**_**R, 43 kDa) and type 2 receptor (D: AT**_**2**_**R, 44 kDa) in aortic tissues**. Representative blots from aortic samples in each group are shown at the top. The bar graph shows protein expression of AT_1_R or AT_2_R relative to internal control ß-actin (45 kDa) from 4 separate experiments in each group. Con: the regular diet group; Cho: the cholesterol-rich diet plus methylthiouracil group; Ros: the group on cholesterol-rich diet plus methylthiouracil and rosiglitazone treatment. Note that rosiglitazone treatment led to the marked reduction in protein expression of AT_1_R, while further increase in protein expression of AT_2_R compared with the Cho group. *P < 0.01 versus Con group; ^‡^*P *< 0.01 versus Cho group.

**Figure 5:**
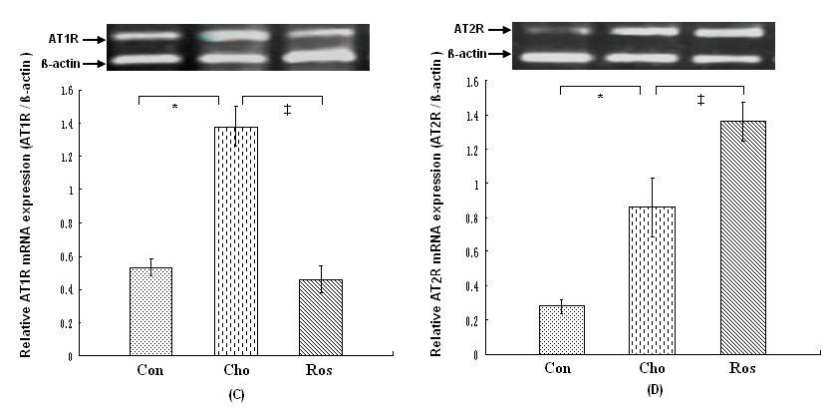
**mRNA expression of angiotensin II type 1 receptor (C: AT**_**1**_**R, 352-bp fragment) and type 2 receptor (D: AT**_**2**_**R, 418-bp fragment) in aortic tissues**. Representative samples of AT_1_R and AT_2_R mRNA from aortic samples in each group are shown at the top. The bar graph shows mRNA expression of AT_1_R or AT_2_R relative to internal control ß-actin (232-bp fragment) from 4 replicate experiments in each group. Con: the regular diet group; Cho: the cholesterol-rich diet plus methylthiouracil group; Ros: the group on cholesterol-rich diet plus methylthiouracil and rosiglitazone treatment. Note that the increase in AT_1_R mRNA caused by the cholesterol-rich diet plus methylthiouracil was markedly suppressed by rosiglitazone treatment (*P *< 0.01). However, mRNA expression of AT_2_R was further increased (*P *< 0.01). *P < 0.01 versus Con group; ^‡^*P *< 0.01 versus Cho group.

Immunohistochemical staining of the proximal aortic sections revealed the presence of AT_1_R and AT_2_R in the endothelial layer and the media in all rats (Figure 3). AT_1_R and AT_2_R showed the lower level of expression in the rats that were fed the regular diet. However, immunoreactivity to both AT_1_R and AT_2_R was clearly visible on the endothelial layer, media, and adventitial tissue in rats on the cholesterol-rich diet plus methylthiouracil, and protein expression of AT_1_R and AT_2_R was dramatically increased compared to rats on a regular diet (Figure 4) (*P *< 0.01). In addition, the rats on the cholesterol-rich diet plus methylthiouracil had much greater levels of AT_1_R mRNA and 3-fold higher AT_2_R mRNA levels than the rats on the regular diet (Figure 5) (*P *< 0.01). Compared with the Cho group, rosiglitazone significantly attenuated AT_1_R immunoreactivity, down- regulated protein and mRNA expression of AT_1_R in the aorta (*P *< 0.01), while further up-regulated protein and mRNA expression of AT_2_R (*P *< 0.01) (Figures 4 and 5).

### Protein and mRNA expression of angiotensin II receptors in VSMCs

**Figure 6:**
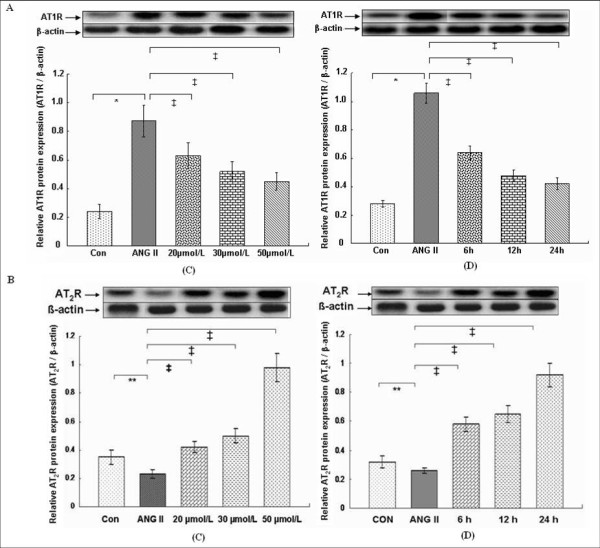
**Effects of rosiglitazone treatment on protein expression of ANG II-induced angiotensin II type 1 receptor (A: AT**_**1**_**R, 43 kDa) and type 2 receptor (B: AT**_**2**_**R, 44 kDa) in vitro cultured VSMCs**. (C): VSMCs were pretreated with 1 μmol/L ANG II for 6 h and subsequently treated for 12 h as follows: Con, ANG II, ANG II plus different concentration rosiglitazones (20, 30, 50) μmol/L; (D): VSMCs were pretreated with 1 μmol/L ANG II for 6 h and subsequently treated with 30 μmol/L rosiglitazone for (6, 12, 24) h, respectively. Representative blots from each experimental group are shown at the top. The bar graph shows protein expression of AT_1_R or AT_2_R relative to internal control ß-actin (45 kDa) from 3 separate experiments in each group. *P < 0.01 versus Con group, ** *P *< 0.05 versus Con group; ^‡^*P *< 0.01 versus ANG II.

**Figure 7:**
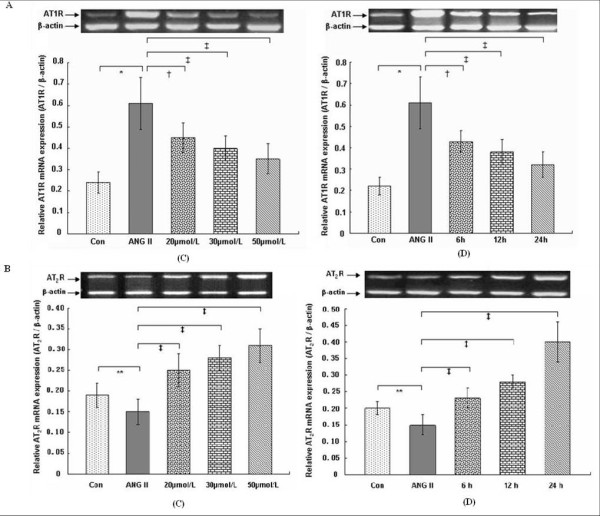
**Effects of rosiglitazone treatment on mRNA expression of ANG II-induced angiotensin II type 1 receptor (A: AT**_**1**_**R) and type 2 receptor (B: AT**_**2**_**R) in cultured VSMCs**. (C): VSMCs were pretreated with 1 μmol/L ANG II for 6 h and subsequently treated for 12 h as follows: Con, ANG II, ANG II plus different concentration rosiglitazones (20, 30, 50) μmol/L; (D):VSMCs were pretreated with 1 μmol/L ANG II for 6 h and subsequently treated with 30 μmol/L rosiglitazone for (6, 12, 24) h, respectively. Representative samples of AT_1_R and AT_2_R mRNA from each experimental group are shown at the top. The bar graph shows mRNA expression of AT_1_R or AT_2_R relative to internal control ß-actin (232-bp fragment) from 3 separate experiments in each group. ** *P *< 0.05 versus Con group, *P < 0.01 versus Con group; ^†^*P *< 0.05 versus ANG II, ^‡^*P *< 0.01 versus ANG II.

In vitro, the protein and mRNA expression of AT_1_R was markedly up-regulated in the VSMCs on ANG II treatment (*P *< 0.01), in contrast, protein and mRNA expression of AT_2_R was down-regulated (*P *< 0.05) (Figures 6 and 7). Compared with the ANG II group, with increasing concentrations of rosiglitazone (20, 30, 50) μmol/L and increasing time (6, 12, 24) h treated with 30 μmol/L rosiglitazone, the protein and mRNA expression of AT_1_R in the VSMCs was markedly attenuated (*P *< 0.05, 0.01). However, protein and mRNA expression of AT_2_R was significantly increased (*P *< 0.01) (Figures 6 and 7).

## Discussion

### Vasculoprotective effects of PPARγ ligand agonists

In the present study, early arterial changes characterized by an accumulation of lipids in intima, VSMC proliferation and migration toward intima were clearly visible in the rats given a high-cholesterol diet plus methylthiouracil for 6 months, although no typical atherosclerotic plaques were observed. The reason that typical atherosclerosis did not develop may be associated with the characteristics of lipid metabolism in the rat *per se*. Thus, it is not easy to produce typical atherosclerosis in rats fed a high-cholesterol diet in the short term, as compared to mice with gene defects or to other animals, such as the rabbit, dog, and monkey. Even so, early arterial changes characterized by an accumulation of lipids, VSMC proliferation and migration toward intima seen in rats on the cholesterol-rich diet plus methylthiouracil were barely observed in the rats treated on a long-term basis with rosiglitazone, which suggested that PPARγ agonists can inhibit atherosclerotic lesions evoked by cholesterol-rich diet and can exert vascular protective effects. Although the amount of animal was small in the current study, this limitation did not affect our observations regarding vascular protective actions of rosiglitazone. Recent study has also demonstrated that PPARγ activation prevented hypertensive remodeling of cerebral arteries and capillary rarefaction as well as improving vascular function without affecting blood pressure [21]. In addition, a common feature of these conditions including cardiovascular diseases, hypertension, dyslipidemia and type 2 diabetes is insulin resistance, which is thought to play a pathogenic role. However, rosiglitazone may improve insulin resistance through inhibiting inflammation of adipose tissue, skeletal muscle and immunologic cells [22,23]. In Zucker diabetic fatty (ZDF) rats, rosiglitazone treatment for 3 weeks restored the endothelial function and adrenergic vasoconstriction, but did not improve the mechanical properties of blood vessel [24]. Although these studies have clearly indicated vasculoprotective effects of PPARγ agonists, the conclusion derived from animal studies in general can not automatically extrapolated to human or clinical studies. However, recently results from meta-analysis studies suggested that rosiglitazone use may be associated with an increase in the risk of myocardial infarction from cardiovascular causes [25,26]. These observations raised questions on the cardiovascular safety of rosiglitazone in the treatment of type 2 diabetes. In fact, the increase in absolute cardiovascular risk after rosiglitazone treatment was very small in these studies on low-risk patients, such as DREAM and ADOPT [27,28]. In the Rosiglitazone Evaluated for Cardiac Outcomes and Regulation of glycemia in Diabetes (RECORD) study, safety analysis suggested nonsignificant changes in cardiovascular morbidity and mortality after rosiglitazone treatment [29]. Furthermore, the results from meta-analyses on pioglitazone and the PROactive (PROspective pioglitAzone Clinical Trial In macroVascular Events) study revealed significant benefits regarding the composite of death, myocardial infarction or stroke. Therefore, the place of TZDs in diabetes treatment strategies still needs further evaluation.

Moreover, VSMCs migration to subintimal space and abnormal proliferation is one of the pathological bases of atherosclerosis. It has been reported that PPARγ agonists inhibit growth factor-induced proliferation and migration of VSMCs [30,31]. PPARγ agonists have also been demonstrated to inhibit cytokine-mediated endothelial cell proliferation and endothelin-1(ET-1) secretion from vascular endothelial cells. The findings of the current study indicated rosiglitazone markedly attenuated ANG II- induced VSMC proliferation partially through down-regulating AT_1_R expression, and up-regulating AT_2_R expression. The effects of PPARγ ligand agonists on VSMCs and vascular endothelial cells are thought to be beneficial in preventing the process of atherosclerosis. Therefore, these results have potentially important implications for optimization of clinical medicine treatments.

### Improvement of lipid profiles by the PPARγ agonist

There is considerable evidence that hypercholesterolemia causes endothelial dysfunction, a prerequisite for atherosclerosis, in conduit vessels and small arteries. Currently available data indicate that PPARγ agonists improve atherosclerosis by ameliorating systemic metabolic risk factors for atherogenesis and inflammatory events [1,32]. The vasculoprotective actions of rosiglitazone can be explained partially by its metabolic regulatory effects in the present study. Rosiglitazone significantly reduced serum TC, TG, and LDL-C induced by a cholesterol-rich diet plus methylthiouracil.

### PPARγ ligand agonists and the renin-angiotensin system

Previous studies have demonstrated that PPARγ ligand agonists inhibit atherosclerotic progression through different molecular mechanisms in humans and animals [33-35]. However, little is known about modulation of RAS components by PPARγ agonists in vasculoprotection. In addition to its pro-hypertensive effects, angiotensin II possesses inflammatory and oxidative effects, and stimulates vascular smooth muscle cell proliferation and migration, which are involved in evolution of atherosclerosis and restenosis [36-38]. PPARγ agonists can inhibit these effects of ANG II by suppression of the ANG II-induced signaling pathway, which suggests that PPARγ agonists have beneficial effects against atherosclerosis and restenosis [39-41]. In the present study, we demonstrated that the reduction of ANG II concentration in the aortic tissues mediated vascular protection of rosiglitazone. The regulatory mechanism concerning ANG II levels in local tissues is still poorly known. It was speculated that the decrease in ANG II levels in aorta may be associated with the suppression of rosiglitazone on angiotensinogen and angiotensin- converting enzyme.

Most of the physiological effects of ANG II, such as regulation of blood pressure and fluid homeostasis, have been attributed to AT_1_R activation. Several studies have also highlighted the important role of AT_1_R in the atherogenic process [15,42,43]. However, the AT_2_R is gaining attention as an opponent to AT_1_R by its counter-regulatory protective roles in the cardiovascular system, e.g. angiotensin type 2 receptor-mediated vasodilation and anti-proliferation [44-47]. Recent studies have also demonstrated the anti- atherosclerotic effects of AT_2_R [48,49]. In our study, PPARγ agonist rosiglitazone markedly suppressed increase in AT_1_R expression in aortic tissues in the hypercholesterolemic rats. In vitro, rosiglitazone significantly attenuated ANG II-induced increase in AT_1_R expression in a concentration-dependent and a time-dependent manner. However, its effect on AT_2_R expression was different, with rosiglitazone further up-regulating AT_2_R expression in the aortic tissues, and suppressing ANG II-induced decrease in AT_2_R expression in a concentration-dependent and a time-dependent fashion. Based on these findings, the beneficial effects of PPARγ agonists on vessels are mediated at least partially by the reduction in local tissue angiotensin II, attenuation of AT_1_R expression and increase in AT_2_R expression although the exact mechanism of PPARγ agonist-mediated regulation of RAS components is still unclear. It has been reported that PPARγ agonist-mediated modulation of AT_1_R has been associated with suppression of the activity of the AT_1_R promoter [50].

In conclusion, the present study demonstrated that PPARγ agonist rosiglitazone attenuated ANG II-induced VSMC proliferation in vitro and early atherosclerotic formation evoked by cholesterol-rich diet in vivo. These vasculoprotective effects of rosiglitazone were mediated at least partially by the improvement of lipid profiles, reduction in local tissue ANG II concentration, down-regulation of AT_1_R expression and up-regulation of AT_2_R expression both at the mRNA and protein levels. However, further studies are necessary to elucidate the molecular mechanism of regulation of RAS components by PPARγ agonists in vascular protection.

## Conflicts of interest

The authors had no conflicts of interest to declare in relation to this article.

## Authors' contributions

LR was responsible for the overall design, drafted and revised the manuscript, as well as participated in the molecular genetic studies. NL conceived of the study, participated in its design and coordination. HZ carried out the culture and treatment of vascular smooth muscle cell. YJL participated in the animal studies and tissue preparation. YZL carried out the measurement of angiotensin II and serum lipids. RT carried out the immunoassays and MTT colorimetric assay. ZS carried out the molecular biological studies and performed the statistical analysis. All authors read and approved the final manuscript.
